# Destroyed Melanocytes Manifested with Poliosis and Halo Nevi in Children: Proposed Immunopathophysiological Mechanisms with Emphasis on Hair Follicle Immune Privilege

**DOI:** 10.3390/clinpract16100184

**Published:** 2026-10-07

**Authors:** Vedrana Bulat, Zrinjka Paštar, Reinhart Speeckaert, Dragana Marušić, Lucija Zanze, Liborija Lugović-Mihić

**Affiliations:** 1 Department of Dermatology and Venereology, Sestre Milosrdnice University Hospital Centre, Vinogradska cesta 29, 10000 Zagreb, Croatia; veckybulat@gmail.com (V.B.); draganamarusic1@gmail.com (D.M.); liborija@sfzg.hr (L.L.-M.); 2 Department of Health, University of Zadar, Splitska 1, 23000 Zadar, Croatia; 3 Department of Dermatology, Ghent University Hospital, 9000 Ghent, Belgium; reinhart.speeckaert@uzgent.be; 4 Family Physician Office, 10000 Zagreb, Croatia; lucijazanze15@gmail.com; 5 School of Dental Medicine, University of Zagreb, 10000 Zagreb, Croatia

**Keywords:** halo nevus, poliosis, immune privilege, immune response, vitiligo, immune privilege collapse, depigmentation, melanocyte destruction

## Abstract

Both poliosis (circumscribed depigmentation of the hair in any area of the body) and halo nevi (a melanocytic nevus which acquires a surrounding depigmented rim) are characterized by depigmentation driven by a T-cell-mediated loss of functional melanocytes within the epidermis and hair bulbs. Halo nevi and poliosis are a relatively frequent phenomenon occurring in 1% of the children and adolescents with an average age of onset of 15 years and has no gender predisposition. Both can be associated with vitiligo and are occasionally seen together as the same cytotoxic immune response (specifically CD8+ T cells) that destroys nevus melanocytes which can attack the same melanocyte-specific targets surrounding epidermal and follicular melanocytes. While poliosis is frequently seen on the scalp, halo nevi on the scalp have been scarcely reported in the literature. The timing of occurrence of the destruction of both epidermal and follicular melanocytes may be supported by a case of a 13-year-old Caucasian male with three halo nevi on the trunk which preceded the development of four scalp patches of poliosis. This may indicate that the halo nevi on the trunk induced a temporary systemic autoimmune reaction during the halo phenomenon, collaterally destroying melanocytes at distance on the scalp causing poliosis.

## 1. Introduction

Melanocyte destruction underlies a heterogeneous group of acquired pigmentary disorders characterized by partial or complete depigmentation of the skin and hair. Among these conditions, vitiligo represents the prototypical chronic, autoimmune melanocytopenic disorder in which autoreactive immune responses target epidermal melanocytes, leading to progressive depigmented macules and patches [1,2,3,4,5,6]. Apart from vitiligo, in which cell-mediated and humoral immune responses contribute to melanocyte destruction, acquired melanocyte loss may also occur in other forms of leukoderma and in certain inflammatory skin reactions. In chemically induced leukoderma, for example, occupational or cosmetic chemicals may exert direct melanocytotoxic effects, resulting in depigmentation [7,8,9,10,11,12,13]. Melanocyte-directed immune mechanisms have also been implicated in halo nevi (a centrally placed melanocytic nevus that becomes surrounded by a 1- to 5 mm depigmented rim, ultimately resulting in the complete regression of the nevus), in which the immune system destroys melanocytes in the mole and surrounding skin [14,15,16]. In addition, melanocyte destruction in the skin, eyes and ears is also seen in Vogt–Koyanagi–Harada disease [17].

Poliosis circumscripta is defined as localized depigmentation of hair resulting from selective reduction or loss of follicular melanocytes in a group of follicles in any hairy area of the body, particularly the scalp [18]. Poliosis may result from either congenital abnormalities of melanocyte development and neural crest migration or acquired loss of follicular melanocytes secondary to autoimmune, inflammatory, toxic, or neoplastic processes. It is most frequently associated with acquired inflammatory or autoimmune disorders, particularly vitiligo and halo nevi [19,20,21,22,23,24].

The aim of this mini-review is to summarize current immunopathophysiological mechanisms underlying melanocyte destruction with particular emphasis on hair follicle immune privilege collapse and follicular melanocyte autoimmunity. In addition, we discuss an illustrative pediatric case of halo nevi-associated poliosis, which may support the concept of transient systemic melanocyte-directed immune activation extending beyond the primary halo phenomenon.

## 2. Methods

A systematic literature search was performed using the PubMed database to identify relevant publications concerning poliosis circumscripta, halo nevi, halo nevi-associated leukoderma, vitiligo, and melanocyte-targeted inflammatory disorders. The search focused on review articles, retrospective and prospective studies addressing the relationship between depigmentation of hair and skin, follicular melanocyte involvement, and halo phenomena. The search strategy employed the following keywords and a combination of keywords, including: “poliosis circumscripta”, “halo nevus”, “halo nevi-associated leukoderma”, “vitiligo”, “depigmentation”, “follicular melanocytes”, “melanocyte destruction”, and “halo phenomenon”. Articles published in English and considered clinically or pathogenetically relevant to the topic were reviewed. Particular attention was given to studies discussing the relationship between epidermal and follicular melanocyte loss, inflammatory mechanisms underlying halo nevi, and the association of poliosis with vitiligo and melanocytic lesions.

Clinical and dermoscopic findings from the present case were analyzed and compared with data available in the literature. In addition, two comparative tables were created to summarize the distinguishing clinical characteristics and proposed pathogenetic relationships among halo nevi-associated leukoderma, halo nevi-associated poliosis, and non-segmental vitiligo.

## 3. Pathogenesis of Poliosis

A comprehensive understanding of poliosis requires insight into hair follicle biology. The hair follicle is a complex structure comprising multiple compartments, including the hair bulb, hair matrix, and bulge region. The hair bulb contains metabolically active melanocytes closely associated with matrix keratinocytes, facilitating melanin synthesis and transfer during the anagen phase of the hair cycle, highlighting the critical role of melanocytes in hair pigmentation [24]. The bulge region of the hair follicle primarily acts as a reservoir for melanocyte stem cells (McSCs) and melanocyte progenitor cells [24]. Melanocyte stem cells (McSCs) are quiescent, unpigmented cells that reside directly in the bulge alongside epithelial stem cells and remain dormant until a new hair growth cycle begins. Melanocyte progenitor cells are early-stage, partially differentiated descendants of the stem cells. When the hair cycle shifts into the active growth phase (anagen), the McSCs in the bulge provide a fresh supply of progenitors, which migrate downward, mature into active melanocytes in the hair bulb, and produce pigment for the hair shaft [24]. Beyond their structural and regenerative roles, these compartments contribute to the establishment of a unique immune environment.

Hair follicles exhibit a unique form of immune privilege, particularly during the anagen phase of the hair cycle, which protects melanocytes from autoimmune-mediated damage. This state is characterized by absent or greatly reduced intra-follicular antigen presentation via major histocompatibility complex (MHC) class I and II molecules and the presence of local immunosuppressive mediators, including α-melanocyte stimulating hormone (α-MSH), transforming growth factor-β (TGF-β), indoleamine 2,3-dioxygenase (IDO), and IL-10, which contribute to the inhibition of T-cell activation within the follicular microenvironment [25]. Under physiological conditions, this privileged state effectively shields melanocytes from recognition by cytotoxic CD8+ T cells and helper CD4+ T cells. However, when immune privilege collapses, MHC molecule expression increases and immune cells gain access to previously protected antigens, allowing autoreactive T cells to recognize and target follicular melanocytes [26]. This collapse may be triggered by oxidative stress, local cytokine imbalances or inflammatory signaling, thereby initiating autoimmune-mediated melanocyte destruction and contributing to conditions such as poliosis, vitiligo, and halo nevus.

Oxidative stress represents an additional contributing factor to melanocyte dysfunction and immune activation. During melanogenesis, melanocytes generate reactive oxygen species (ROS) as physiological by-products. When ROS production exceeds the capacity of endogenous antioxidant defenses, including enzymes such as catalase and glutathione peroxidase, melanocytes experience oxidative damage affecting DNA, proteins, and lipids [27]. This imbalance compromises melanocyte viability and can lead to apoptosis, which manifests in vitiligo as depigmented skin patches. Although poliosis has not been specifically studied in this context, similar mechanisms are hypothesized to contribute to localized hair depigmentation observed in pediatric patients with poliosis, halo nevi, or vitiligo.

In addition to direct cellular injury, oxidative stress promotes the release of damage-associated molecular patterns (DAMPs) from stressed melanocytes and surrounding keratinocytes. These DAMPs serve as endogenous danger signals recognized by pattern recognition receptors on innate immune cells, including dendritic cells and natural killer cells, resulting in innate immune activation [28]. This activation promotes antigen presentation and primes adaptive immune responses, particularly involving CD8+ T lymphocytes, linking oxidative stress to immune-mediated melanocyte destruction [29].

A central pathway in melanocyte-targeted autoimmunity involves IFN-γ-mediated activation of the JAK–STAT signaling cascade. IFN-γ binds to its receptor on melanocytes, keratinocytes, and dermal fibroblasts, activating Janus kinases 1 and 2 (JAK1 and JAK2), resulting in phosphorylation and nuclear translocation of STAT1. This signaling induces transcription of chemokines such as CXCL9 and CXCL10, which play a key role in the recruitment of CXCR3+ CD8+ T cells to affected tissues [4,30,31]. These cytotoxic T cells contribute to targeted melanocyte destruction and amplification of local inflammation. While this pathway is well established in vitiligo, it likely represents a shared immunological mechanism underlying follicular melanocyte loss in poliosis [30,31].

Fibroblasts, when stimulated by IFN-γ, secrete CXCL9 and CXCL10, enhancing the recruitment of cytotoxic T cells and perpetuating melanocyte loss [32]. This demonstrates that non-immune stromal cells actively contribute to follicular autoimmunity.

Poliosis in children arises from a complex, multifactorial autoimmune process. Key components include oxidative stress-induced melanocyte injury, collapse of follicular immune privilege, IFNγ-driven activation of the JAK–STAT pathway, chemokine-mediated recruitment of CD8+ T cells, cytotoxic destruction of follicular melanocytes, and amplification by dermal fibroblasts. Its association with halo nevus and vitiligo underscores a common autoimmune pathophysiology, highlighting the interplay between melanocytes, keratinocytes, fibroblasts, and immune cells in mediating hair depigmentation. Taken together, vitiligo, halo nevi, and poliosis are closely related pigmentary conditions characterized by immune-mediated destruction of melanocytes and share a similar pathogenesis driven by the convergence of genetic, autoimmune, and metabolic factors, with psychological stress potentially acting as a significant trigger for disease and systemic comorbidity.

## 4. Genetic and Acquired Conditions Associated with Poliosis

Poliosis may be associated with various genetic conditions and acquired disorders (Table 1). Developmental absence of melanocytes and immune-mediated destruction of melanocytes represent biologically distinct pathways leading to a similar clinical endpoint (poliosis). Thus, it is possible to see poliosis in genetic conditions such as piebaldism, Waardenburg syndrome, tuberous sclerosis, Tietze syndrome, neurofibromatosis type 1, Marfan syndrome, and prolidase deficiency. Also, poliosis may be associated with acquired disorders such as Vogt–Koyanagi–Harada syndrome, Alezzandrini syndrome, alopecia areata, sarcoidosis, blepharitis, drug-induced skin reactions, trigeminal autonomic cephalalgia, postherpetic infection, trauma from repetitive plucking, post-irradiation effects, and solitary neurofibromas [17,18,19,33,34,35]. However, the most common poliosis association is with vitiligo and various types of melanocytic lesions, including both benign (congenital and acquired nevi) and malignant neoplastic entities (mainly melanoma) [21,36,37,38,39,40].

Regarding the occurrence of depigmentation associated with melanocyte loss, van Geel et al. reported that some patients with halo nevi subsequently developed vitiligo, whereas in others vitiligo was already present, suggesting a possible link between halo nevi and vitiligo [38]. This observation underscores the clinical correlation between halo nevi, vitiligo, and localized hair depigmentation, implying that autoimmune responses can target follicular melanocytes specifically.

Halo nevi are a relatively common phenomenon, occurring in approximately 1% of the population [41]. These lesions are common in children and young adults, with an average age of onset of 15 years, and have no gender predisposition [42]. The development of multiple halo nevi is observed in 25–50% of patients with halo nevi [14].

Halo nevi exemplify follicular melanocyte-targeted autoimmunity. Histologically, lymphocytic infiltrates surround nevus cells and are often associated with adjacent hair follicle poliosis. The clinical co-occurrence of halo nevi, vitiligo, and poliosis suggests a shared autoimmune etiology in which cytotoxic lymphocytes recognize melanocyte antigens in both epidermal and follicular compartments [41].

## 5. Experience with Poliosis from Pediatric Clinical Practice

**Figure 1:**
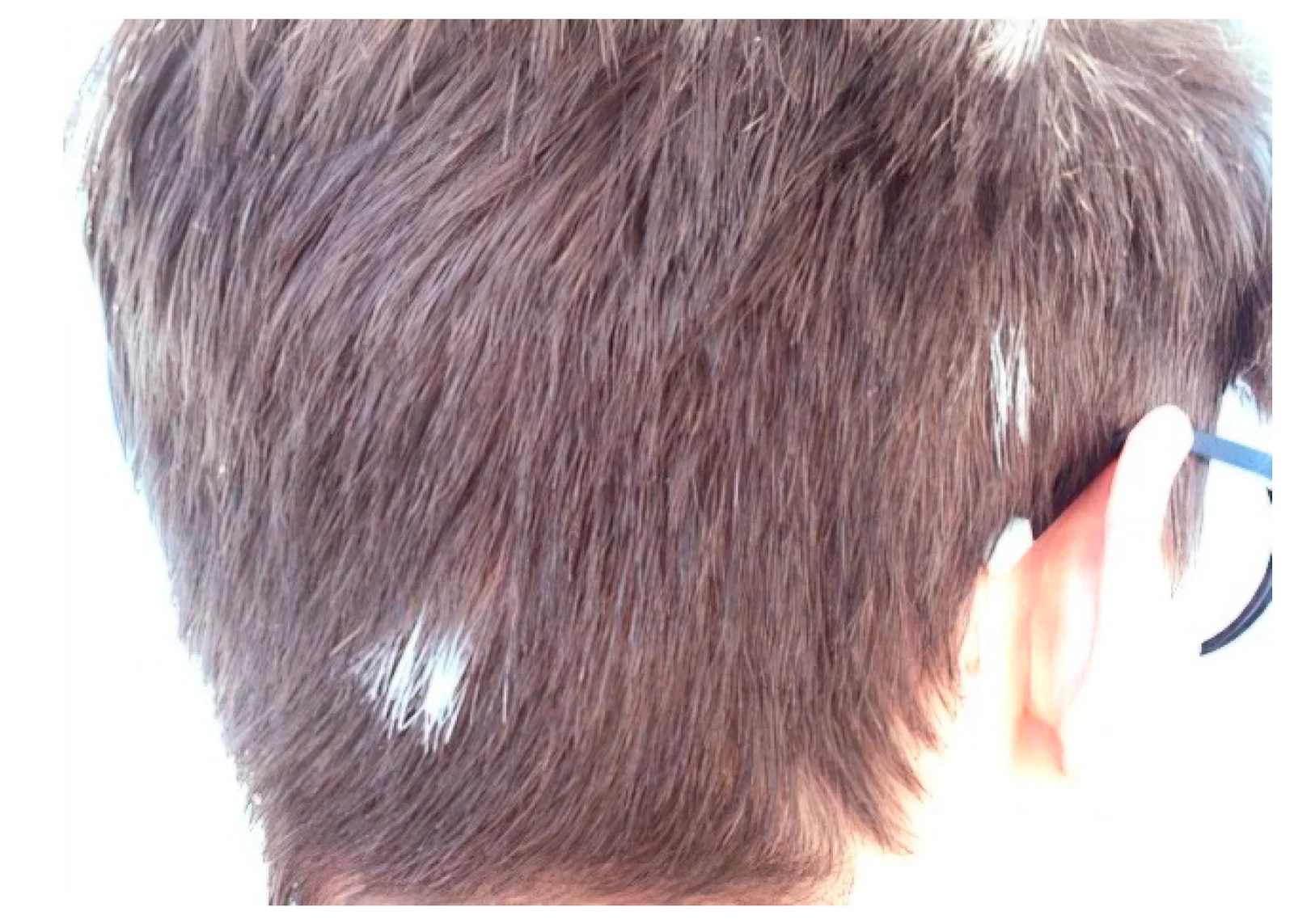
Clinical overview of the scalp showing the distribution of multiple areas of poliosis.

**Figure 2:**
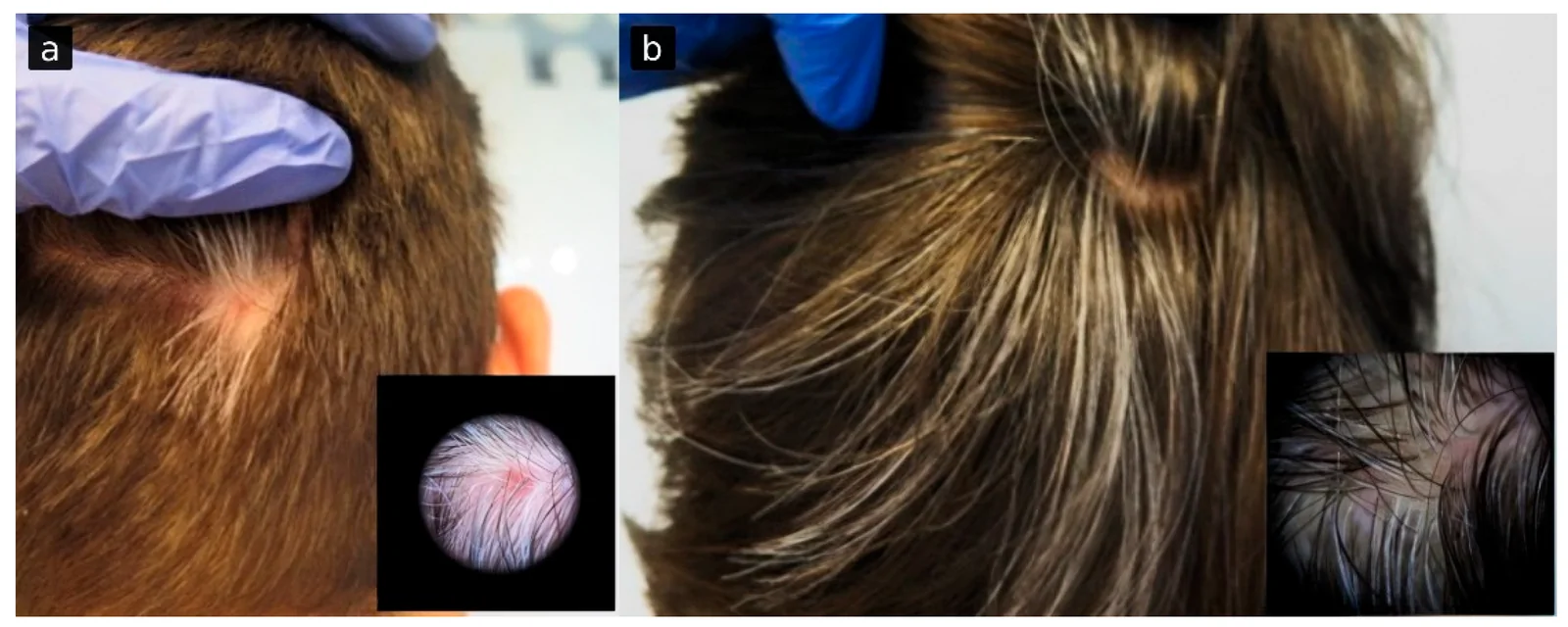
Detailed clinical and dermoscopic images from the same patient shown in Figure 1, obtained from different viewpoints and at greater magnification. In the clinical close-up photographs, the hair was manually parted under macro illumination to visualize the scalp surface. (**a**) Round area of poliosis on the right side of the scalp associated with a clinically and dermoscopically identifiable halo nevus; inset: dermoscopic image of the corresponding lesion. (**b**) Irregularly shaped area of poliosis on the left side of the scalp without a clinically or dermoscopically visible nevus; inset: dermoscopic image of the corresponding area.

Poliosis associated with genodermatoses usually presents with a single patch of white hair on the scalp from birth. However, in our clinical practice, one patient (a 13-year-old Caucasian boy) had acute onset of four acquired patches of white hair on his scalp, without involvement of the eyebrows, eyelashes, or other hairy areas of the body. The patient, with Fitzpatrick skin phototype II and fewer than 10 common nevi, presented to our hospital for examination of his nevi. Depigmentation around three nevi on the trunk had begun one year earlier, while the white patches of hair had appeared ten months earlier, first on the right side of the scalp, followed by two additional white patches on the left side of the scalp. Dermatological examination revealed three halo nevi: one on the upper anterior trunk, dermoscopically characterized by uniform brown globules surrounded by a typical depigmented whitish halo. The other two halo nevi were located on the lower abdomen, with complete clinical and dermoscopic disappearance of pigment and visible linear vessels. On the scalp, there were four areas of poliosis (Figure 1). In two areas on the right side of the scalp, dermoscopic examination revealed halo nevi with round poliosis (Figure 2a). In the other two areas of poliosis on the left side of the scalp, no clinically or dermoscopically visible nevi or remnants of vessels were found (Figure 2b), and these areas of poliosis were irregularly shaped. The eyebrows, eyelashes, and other hairy areas of the body were not involved. All other nevi on the body were clinically and dermoscopically common, with a globular pattern. There was no personal or family history of autoimmune disease, halo nevi, or vitiligo. There was also no history of medication use or trauma and no evidence of the Koebner phenomenon, mucosal involvement, skin depigmentation, nystagmus, iris heterochromia, or facial dysmorphism. An audiogram revealed normal hearing thresholds across all tested frequencies. He did not recall any past sunburn, but mentioned extensive tanning a year before the onset of his halo nevi.

Wood’s light examination of the whole body did not reveal additional depigmentation. Serological tests for autoimmunity, including antinuclear, antithyroperoxidase, and antithyroglobulin antibodies, were negative; ferritin, UIBC, and TIBC levels were normal. Vitamin D level was low, and supplementation was prescribed. Lyme disease serology was negative. Testing for HLA alleles positively associated with vitiligo (HLA-DR3, -DR4, and -DR53) was negative. In our patient, halos developed around three globular, “early-onset” nevi on the upper body, with two round areas of poliosis on the right side of the scalp around common flat nevi. However, two additional areas of poliosis were irregularly shaped on the left side of the scalp, without nevi or remnants of nevi. The halo nevus on the trunk was removed at the request of the patient and his parent. Histopathological examination of the surrounding depigmented halo showed a clear lymphocytic infiltrate. Immunohistochemical staining demonstrated CD8+, CD4+, and CD1a+ cells, while relatively sparse FoxP3+ regulatory T cells were identified. These findings were considered most consistent with halo nevi-associated poliosis.

No systemic treatment was initiated because the lesions were localized and clinically stable. The patient underwent regular dermatological follow-up. During two years of observation, no new halo nevi, vitiligo lesions, or additional areas of poliosis developed. Existing lesions remained stable without clinically significant progression or spontaneous repigmentation. Longer follow-up was recommended because progression toward vitiligo or development of additional melanocyte-targeted immune phenomena could not be completely excluded.

## 6. Current Knowledge on Poliosis and Associated Conditions and Questions Related to Its Features

Halo nevi on the scalp have been scarcely reported in the literature. As a result, they are often confused with vitiligo and alopecia areata. Depigmentation of scalp hairs is uncommon in non-segmental vitiligo, in contrast to segmental vitiligo and halo nevi [43]. The onset is usually earlier in segmental vitiligo and halo nevi and rapidly involves the follicular melanocyte reservoir, resulting in hair whitening. The collapse of anagen hair bulb immune privilege is an essential prerequisite for the development of poliosis [26,44,45].

The relative immune privilege of the follicular melanocyte reservoir could explain why hairs on the affected skin remain pigmented initially in non-segmental vitiligo [45]. The difference between segmental vitiligo and halo nevi may be due to a cytotoxic immune response against different melanocyte-specific antigens. The present case demonstrates several distinct clinical and immunogenetic features suggesting that halo nevi-associated poliosis may be pathophysiologically distinct from vitiligo-associated poliosis (Table 2). First, the patient lacked generalized, progressive skin depigmentation, mucosal involvement, and personal or familial autoimmune predisposition, which stands in stark contrast to the polygenic, systemic autoimmune background typical of vitiligo. Second, the depigmented lesions remained localized and clinically stable during follow-up. Third, vitiligo-associated HLA susceptibility loci were absent, further solidifying the distinct genetic architecture driving this presentation.

Particularly interesting was the presence of two irregularly shaped depigmented scalp areas without clinically or dermoscopically identifiable melanocytic remnants. This finding suggested that the follicular depigmentation may not simply represent regression of halo nevi on the scalp but rather collateral melanocyte destruction occurring during a broader immune response associated with the halo phenomenon on the trunk. Although difficult to confirm with certainty in our patient, two areas of irregular depigmentation without remnants of a regressed nevus suggested that these depigmentations were an epiphenomenon of the halo nevi process on the trunk. The morphology of the scalp lesions in our patient differed fundamentally from classic vitiligo-associated poliosis. While poliosis associated with vitiligo typically presents insidiously as a single, circumscribed patch of white hair due to the gradual, chronic depletion of the epidermal–follicular melanin unit, our patient experienced the abrupt and synchronous development of four distinct, acquired patches of white hair. This explosive, multi-focal onset implies an acute, high-intensity cytokine storm rather than a low-grade, chronic autoimmune progression.

Our histopathological and immunohistochemical findings supported melanocyte-targeted immune activation characterized by a profound predominance of cytotoxic inflammatory infiltrates and a relative reduction of regulatory T cells (Tregs). The presence of large numbers of antigen-presenting Langerhans cells in the depigmented rim of the halo nevus in our patient suggests a failure of tolerance to self-melanocytic antigens, effectively priming the adaptive immune system against melanocytes. The observed predominance of CD8+ inflammatory infiltrates together with sparse FoxP3+ regulatory T cells further supports loss of tolerance toward melanocytic antigens. Although these findings are consistent with an immune-mediated melanocyte-directed process and correlate with previous observations in halo nevi and vitiligo, they are not specific and do not allow definitive conclusions regarding the precise immunopathogenic mechanisms in our patient. Our hypothesis is that the halo nevi on the trunk induced a temporary systemic immune reaction during the halo phenomenon, collaterally destroying melanocytes in distant areas of the scalp. One possible explanation is that, if a sufficiently potent systemic cytotoxic immune response was triggered during the halo phenomenon in multiple halo nevi, it could potentially extend to the follicular compartment and affect the resident McSCs. However, this mechanism was not directly investigated in the present patient and therefore remains speculative.

Once these stem cells are eradicated, the hair follicle completely loses its regenerative potential. Although the nature of the halo phenomenon is transient, repigmentation of poliosis remains impossible because the biological source required to repopulate the follicle with functional melanocytes has been entirely depleted. This irreversible destruction of the stem cell niche elucidates the clinical manifestation of permanent poliosis observed in our patient.

Van Geel et al. previously proposed the concept of halo nevi-associated leukoderma (HNAL) to describe several patients with multiple halo nevi and discrete skin depigmentations distant from halo nevi, but these patients did not have poliosis [14]. In our patient, the halo nevi and the areas of irregularly shaped poliosis developed over a two-month period, with five halo nevi that did not progress in size or number over the following two years. Unlike halo nevi-associated leukoderma, which is characterized by distant vitiligo-like cutaneous depigmentation in patients with multiple halo nevi, our patient had no depigmented skin lesions outside the immediate halo areas. Follow-up is needed to monitor the patient for the development of additional halo nevi, poliosis, or even vitiligo, as well as for the potential repigmentation of depigmented hair. In addition, van Geel et al. suggested that this epiphenomenon could be a temporary autoimmune process directly linked to the development of halo nevi and could represent collateral damage resulting from skin immunosurveillance rather than vitiligo [14]. Moreover, the clinical impact of this is important, as the evolution of HNAL is less progressive. The lesions are moderately demarcated, asymmetric, and limited in size, with involvement of various uncommon body areas, and the age of onset is lower compared with vitiligo [14,37]. Our observations raise the possibility that a similar phenomenon may occur within hair follicles. Although this finding is based on a single patient and therefore cannot establish a distinct clinicopathological entity, it generates the hypothesis that halo nevi-associated poliosis may represent a previously unrecognized clinical phenotype deserving further investigation.

Moreover, the characteristics of this patient with halo nevi-associated poliosis differed from those of scalp halo nevi. Scalp halo nevi with local regression were considered in the differential diagnosis; however, two irregular areas of poliosis lacked clinically or dermoscopically detectable nevi or vascular remnants suggestive of completely regressed nevi. Alopecia areata was excluded because there was no hair loss and the scalp examination revealed isolated poliosis. Waardenburg syndrome was considered unlikely because the patient had normal hearing, and no iris heterochromia, dystopia canthorum, or characteristic facial features. Vogt–Koyanagi–Harada disease was also considered unlikely because there were no ocular, auditory, or neurological manifestations. The history and clinical findings did not support other congenital or inflammatory causes.

The presentation also differed from vitiligo-associated poliosis because there was no typical segmental or non-segmental vitiligo pattern, no progression during the two-year follow-up, and no personal or family history of autoimmune disease. These distinctions suggest that the observed pattern may represent an unusual phenotype within the spectrum of halo-associated melanocyte-directed immune phenomena. However, additional cases are required before it can be regarded as a distinct clinicopathological condition.

The timing of poliosis onset suggested a direct relationship with the halo phenomenon. Unlike the HNAL entity, no vitiligo-like depigmentations were present in areas other than the scalp. Our patient also does not express specific vitiligo-associated autoimmune susceptibility genes. In some patients, halo nevi may trigger the development of additional depigmentations whose distribution, extent, and prognosis do not align with vitiligo [14]. Hypothetically, the localized depigmentation of hairs could result either from halo nevi on the scalp with complete disappearance of the nevi or from a systemic immune response associated with halo nevi on the trunk. During the acute phase of the halo phenomenon, the CD8+/FoxP3+ ratio may shift dramatically in favor of cytotoxicity. Both local and systemic Tregs are temporarily dysfunctional due to the profound failure of peripheral tolerance triggered by antigen-presenting Langerhans cells. This transient yet massive absence of immune suppression creates a permissive window that enables the rapid, non-selective destruction of melanocytes at distant anatomical sites, such as the scalp in our patient. The immunohistochemical findings should be interpreted with caution because they were obtained from a single lesion and provide only indirect evidence of the underlying immune mechanisms. Additional molecular and functional studies would be required to validate the proposed pathogenetic model.

## 7. Conclusions

Recognizing the different phenomena leading to poliosis is crucial for gaining insight into the pathophysiology of immune reactions against the follicular melanocyte reservoir and for guiding future research. We suggest differentiating halo nevi-associated poliosis from scalp halo nevi and from poliosis associated with classical vitiligo, based on distinct clinical characteristics. Although this single observation cannot establish a distinct clinicopathological entity, it expands the spectrum of possible immune-mediated melanocyte disorders and raises the hypothesis that halo nevi-associated poliosis may represent a distinct clinical phenotype. Further studies, including additional well-characterized patients and longer-term follow-up, are required to determine whether this observation represents a reproducible clinicopathological pattern and to clarify the underlying immunopathogenic mechanisms.

## Figures and Tables

**Table 1:** Genetic and acquired conditions associated with poliosis.

Disease	Clinical Features	Etiology	Pathogenesis	Histopathology	Prognosis
**Vitiligo**	Non-scaly; chalky-white macules and patches from selective melanocyte loss; prevalence 0.36%; no sex or racial predisposition; onset in the first three decades (1/3 ~10 y; 2/3 ~30 y). Strong associated comorbidities: thyroid disease (15–25%); alopecia areata (3–8%); atopic dermatitis (10–30%); depression (12–50%) [1,2,3,4,5]. NSV: symmetrical patches; Koebner phenomenon in 15–70%; expansion over time. SV: unilateral; segmental distribution; early onset (median 14 y); self-limited; stabilizes within 6–24 months; poliosis in up to 50% of affected areas [1,2,6,7].	Multifactorial autoimmune disease; 75–83% of disease risk is genetic; ~20% environmental; polygenic inheritance pattern. Monozygotic twin concordance ~23% [3,39,40]. GWAS identified >50 susceptibility loci; key loci include HLA; PTPN22; TYR; GZMB; RERE. >50% of loci involve immunoregulation and T-cell signalling [1,3,22,39]. Environmental triggers: UV radiation; phenolic compounds; mechanical trauma; pregnancy; psychological stress (trigger in ~50%) [1,3,22].	ROS accumulation → mitochondrial dysfunction + ER stress → DAMP release → DC maturation → innate immune priming and antigen presentation → IFN-α; IFN-γ release [22]. Effector phase: CD8+ CTLs target Melan-A/MART-1; tyrosinase; gp100; IFN-γ → keratinocyte CXCL9/CXCL10 → CXCR3+ T-cell recruitment (self-amplifying loop). CD8+ TRM persist in lesional skin (maintained by IL-15 and TGFβ) → principal relapse mechanism [22,23]. Treg dysfunction → unopposed CD8+ autoreactivity; more pronounced in NSV than SV. Novel melanocyte death modalities: necroptosis; ferroptosis; oxeiptosis. E-cadherin loss via MMP-9 (IFN-γ/TNF-α) → transepidermal melanocyte loss. Impaired WNT–β-catenin signalling (IFN-γ) → compromised repigmentation [22].	Established lesion: Absent or near-absent basal epidermal melanocytes. Inflammatory infiltrate in up to 48% of biopsies; active disease shows lichenoid interface dermatitis with CD8+-predominant perivascular and periadnexal infiltrate at the perilesional margin [1,3]. Perilesional skin: enlarged; vacuolated melanocytes with melanin-filled elongated dendrites; vacuolar DEJ changes with mononuclear infiltrate in clinically normal adjacent skin—indicating subclinical disease activity beyond the visible lesion boundary [1]. Clinically unaffected skin: Basal melanocyte density ~25% lower than healthy controls; transepidermal melanocyte loss (detachment and upward migration); stratum corneum thickening [3,22].	NSV: chronic; relapsing; spontaneous repigmentation rare without treatment. SV: stabilises within 6–24 months [6]. Intact follicular reservoir (no leukotrichia) is the key favourable indicator → perifollicular repigmentation after phototherapy (NB-UVB). Follicle-poor sites (lips; distal digits; bony prominences) respond poorly. ~50% relapse at the same site within 1 year of cessation [3].
**Halo nevus (Sutton’s nevus)**	Benign melanocytic nevus with symmetrical depigmented halo (0.5–5 cm); prevalence ~1%; mean onset ~15 years; no sex or racial predilection; predominantly trunk and head/neck; 25–50% have multiple lesions [15,16,21]. Four stages: I—depigmented rim; II—central pigment loss + rim; III—central nevus absent; IV—spontaneous repigmentation. Course >10 years; complete regression ~50%. The halo phenomenon also occurs around Spitz, blue, and congenital nevi, as well as melanoma; new onset in adults aged >40 years requires melanoma exclusion [15,16]. ~20% develop associated vitiligo; younger onset carries greater risk; Koebner phenomenon and family history of vitiligo further increase this risk [16,21].	Cell-mediated immune response against nevomelanocyte antigens. Genetic predisposition: higher frequency with increased nevus count or personal/family history of vitiligo; predisposition to systemic autoimmune diseases (especially Hashimoto’s thyroiditis); familial cases reported [15,16]. Transcriptomics: IFN-related pathway enrichment overlapping with vitiligo signatures; specific HLA-I–restricted antigenic targets remain unknown. Drug-induced: documented with atezolizumab and ipilimumab [15,21].	Innate priming: oxidative stress → UDP-glucose (DAMP) → DC activation → T-cell priming; IFN-γ/CXCL9/CXCL10 recruit effectors; HLA-I upregulated; pyroptosis and ferroptosis co-activated [15]. Effector phase: CD8+ CTLs (~80% of infiltrate); increasing with regression stage; express granzyme B; perforin; FasL; granulysin; CD4+ cells provide auxiliary support. Regulatory control: IFN-γ → PD-L1 induction—negative feedback limiting cytotoxicity; FOXP3+ Tregs present in all stages [15,16,21].	Arises in association with compound or intradermal nevi. Stage-dependent infiltrate: I—intact nests; ~50% CD3+/~25% CD4+; II—S-100+ LCs increase; mild nuclear atypia; III—CD8+ (granzyme B+; perforin+; FasL+) dominate; complete melanocyte loss; IV—repigmentation; epidermal flattening; superficial neovascularisation. No dermal fibrosis [15,16].	Benign; self-limited; complete central nevus regression in ~50% over >10 years; followed by possible spontaneous halo repigmentation; atypical evolution (nevus repigmentation without full regression) also reported [15,16]. Multiple halo nevi in adults (>40 yrs) require melanoma workup.
**Chemical leukoderma**	Acquired depigmentation indistinguishable from vitiligo; site-limited 74%; remote 26%; all ages; females predominate. Diagnosis: ≥3 of 4 criteria—(1) acquired vitiligo-like lesions; (2) repeated chemical exposure; (3) macule distribution at exposure site; (4) confetti macules [7,8,9]. Staging: I—contact site; II—local spread; III—systemic spread; IV (“chemical vitiligo”)—continues >1 year post-cessation [7,8,10].	Principal agents are para-substituted phenols/catechols (MBEH; 4-TBP; 4-TBC; 4-TAP; rhododendrol) and PPD—all act as tyrosinase/TYRP-1 substrates generating reactive ortho-quinones selectively toxic to melanocytes. Non-phenolic agents (mercurials; tretinoin; chloroquine) implicated via distinct mechanisms [7,8,11,12].	Direct cytotoxicity: phenols (tyrosine analogues) → tyrosinase/TYRP-1 → ortho-quinones → GSH depletion + ER stress (UPR) → autophagy → apoptosis [7,10,11]. Autoimmune spread: stressed melanocytes → HSP70 (DAMP) → DC activation → Melan-A-specific CD8+ CTL + NK recruitment; IFN-γ → CXCL9/CXCL10 → T-cell recruitment loop; TRAIL upregulation → systemic generalisation. Shares pathogenic spectrum with idiopathic vitiligo; individual genetic factors determine local vs. generalised spread [7,8,10,11].	Established lesions: histologically indistinguishable from vitiligo; no absolute histopathological criteria separate the two [7,8,10]. When spread beyond contact site: complete melanocyte loss; CD8+ T-cell predominance; indistinguishable from NSV. Leukotrichia absent (unlike vitiligo), reflecting follicular immune privilege. [7,10,12].	Exposure cessation is mandatory; repigmentation is perifollicular and gradual, occurring over weeks to months. De novo (contact-site-limited) cases respond better than chemically exacerbated pre-existing vitiligo [7,8]. Stage IV (“chemical vitiligo”): continues >1 year post-cessation; may be irreversible; requires systemic immunomodulation. Early diagnosis is essential to interrupt autoimmune initiation before generalisation [7,8,13].
**Poliosis circumscripta**	Localised patch of depigmented hair in any hair-bearing area. Congenital: piebaldism (white forelock); Waardenburg syndrome (white forelock + sensorineural deafness); tuberous sclerosis (earliest neonatal sign); congenital nevus [18]. Acquired: VKH—poliosis with vitiligo and alopecia (third/chronic phase); vitiligo—up to 25% overall; 48.6% in segmental vitiligo; alopecia areata—whitened regrowing hairs or migratory poliosis without prior hair loss; neoplastic—acquired nevi (halo reaction); NF; melanoma (acute scalp poliosis warrants malignancy exclusion); sarcoidosis; blepharitis; drug-induced (prostaglandin analogs; cetuximab; acitretin; ICI; chloroquine) [18,19].	Congenital: piebaldism → KIT/SLUG/SNAI2 mutations → melanoblast migration failure; Waardenburg → neural crest lineage disruption (melanocytes; auditory neurons; enteric ganglia); TSC1/TSC2 → reduced melanin and melanosomes in follicular bulbs [18,20]. Acquired autoimmune: VKH—T-cell-mediated attack against tyrosinase-family antigens; AA—HF immune privilege collapse → autoreactive CD8+ CTLs. Neoplastic/drug-induced: acquired nevi—CTL cross-reactivity destroys adjacent follicular melanocytes; congenital nevi—non-inflammatory; migration arrest; ICI—disinhibits autoreactive T cells; prostaglandin analogs—reduce melanogenesis via tyrosinase inhibition [17,18,19,33,34].	Two mechanisms share the same endpoint (absent/reduced follicular melanocytes): (1) developmental absence—failed neural crest migration (piebaldism; WS; congenital nevi); (2) acquired destruction—immune; toxic; or inflammatory; follicular architecture preserved (non-scarring) in both [18]. Immune-mediated: CD8+ CTLs + NK cells infiltrate the anagen hair bulb (IFN-γ-driven); AA—bulge stem cells spared; follicular melanocytes selectively lost; sarcoidosis—uniquely humoral (anti-melanocyte antibodies). Neoplastic/drug-induced: acquired nevi—CTL cross-reactivity confirmed by poliosis resolution after excision; neurofibromatosis—anti-neurofibroma T cells cross-react with hair bulb melanocytes; ICI—disinhibited MelanA-specific T cells; prostaglandin analogs—tyrosinase inhibition [18,19,33,34,35].	Absent/reduced DOPA-positive melanocytes in hair bulbs. Hair shafts depigmented; overlying epidermal melanocytes preserved unless concurrent vitiligo is present [18]. Congenital (piebaldism; WS): no infiltrate; follicle intact. TSC: reduced melanin and melanosomes. Acquired nevi: lymphohistiocytic perifollicular infiltrate (CTL-mediated). Congenital nevi: no infiltrate; migration arrest [18]. Autoimmune (AA): peribulbar CD8+ CTL infiltrate with CD4+ T cells; bulge epithelial stem cells spared; follicular architecture preserved (non-scarring). ICI-induced AA-like alopecia: peribulbar infiltrate predominantly CD4+ (scant CD8+) [19,33,34].	Congenital (piebaldism): stable/permanent from birth; rare spontaneous repigmentation possible. WS: prognosis dominated by sensorineural hearing loss (+ Hirschsprung disease in WS4). TSC: poliosis as earliest sign enables early diagnosis—critical for management and genetic counselling [18,20]. VKH: poliosis/vitiligo reversal in ~27% with adequate immunosuppression; early corticosteroids standard of care. AA: JAK inhibitors (baricitinib; ritlecitinib) may restore HF immune privilege. Vitiligo-associated: mirrors vitiligo course. Drug-induced: repigmentation after cessation in some cases. ICI: photoprotection only; positive biomarker for anti-melanoma efficacy. Neoplastic: excision → complete poliosis resolution [17,18,19,35].

Abbreviations: 4-TAP—4-tert-amylphenol; 4-TBC—4-tert-butylcatechol; 4-TBP—4-tert-butylphenol; AA—alopecia areata; ClinROMs—clinician-reported outcome measures; CTL—cytotoxic T lymphocyte; CXCL9/CXCL10—C-X-C chemokine ligand 9/10; CXCR3—C-X-C chemokine receptor 3; DAMP—damage-associated molecular pattern; DC—dendritic cell; DEJ—dermoepidermal junction; DOPA—dihydroxyphenylalanine; ER—endoplasmic reticulum; FasL—Fas ligand; FOXP3—forkhead box protein P3; GSH—glutathione; GWAS—genome-wide association study; HF—hair follicle; HLA—human leukocyte antigen; HMB-45—human melanoma black-45; HSP70—heat shock protein 70; ICI—immune checkpoint inhibitor; IFN-α—interferon-alpha; IFN-γ—interferon-gamma; IL-15—interleukin-15; JAK—Janus kinase; KIT—proto-oncogene receptor tyrosine kinase; LC—Langerhans cell; MART-1—melanoma antigen recognised by T cells 1; MBEH—monobenzyl ether of hydroquinone; MMP-9—matrix metalloproteinase-9; NB-UVB—narrowband ultraviolet B; NCECS—non-cultured epidermal cell suspension; NF—neurofibromatosis; NK—natural killer (cell); NSV—non-segmental vitiligo; PD-L1—programmed death ligand 1; PPD—para-phenylenediamine; PROMs—patient-reported outcome measures; ROS—reactive oxygen species; SBEG—suction blister epidermal grafting; SLUG/SNAI2—snail homolog 2; SV—segmental vitiligo; TGF-β—transforming growth factor beta; TNF-α—tumour necrosis factor alpha; TRAIL—TNF-related apoptosis-inducing ligand; Treg—regulatory T cell; TRM—tissue-resident memory T cell; TSC—tuberous sclerosis complex; TYRP-1—tyrosinase-related protein 1; UDP—uridine diphosphate; UPR—unfolded protein response; UV—ultraviolet; VKH—Vogt–Koyanagi–Harada syndrome; WNT—Wingless-related integration site; WS—Waardenburg syndrome.

**Table 2:** Differences between halo nevi-associated leukoderma, halo nevi-associated poliosis and non-segmental vitiligo.

	Halo Nevi-Associated Leukoderma	Halo Nevi-Associated Poliosis	Non-Segmental Vitiligo
Extent of skin depigmentation	Limited patches	No	Extensive patches
Location of skin depigmentation	Variable; uncommon locations	No	Predilection sites (elbows; knees; dorsal hands and feet; perioral region)
Ability to progress	Non-progressive	Non-progressive	Progressive
Number of halo nevi	Multiple (≥5)	Multiple (≥5)	Usually few (1–3)
Poliosis	No	Multiple	One or none

## Data Availability

The original contributions presented in this study are included in the article. Further inquiries can be directed to the corresponding author.
